# Reducing the virulence of *Pseudomonas aeruginosa* by using multiple quorum-quenching enzymes

**DOI:** 10.1093/jimb/kuad028

**Published:** 2023-09-20

**Authors:** Mst Afroza Khatun, Md Anarul Hoque, Mattheos Koffas, Yan Feng

**Affiliations:** State Key Laboratory of Microbial Metabolism, School of Life Sciences and Biotechnology, Shanghai Jiao Tong University, Shanghai 200240, China; Department of Chemical and Biochemical Engineering, Center for Biotechnology and Interdisciplinary Studies, Rensselaer Polytechnic Institute, Troy, NY 12180, USA; Department of Chemical and Biochemical Engineering, Center for Biotechnology and Interdisciplinary Studies, Rensselaer Polytechnic Institute, Troy, NY 12180, USA; State Key Laboratory of Microbial Metabolism, School of Life Sciences and Biotechnology, Shanghai Jiao Tong University, Shanghai 200240, China

**Keywords:** *Pseudomonas aeruginosa*, Quorum sensing, Quorum quenching, AidC, QQ-2

## Abstract

The emergence of multidrug-resistant *Pseudomonas aeruginosa* in healthcare settings poses a tremendous challenge to traditional antibiotic therapy. *Pseudomonas aeruginosa* utilizes quorum sensing (QS) to coordinate the production of virulence factors and the formation of drug-resistant biofilms. QS is mediated by signal compounds produced by *P. aeruginosa* as well as signal molecules produced by other non-pseudomonad bacteria. A potential strategy to prevent bacterial pathogenicity is utilizing enzymes to interfere with QS. Here, we used AidC, a quorum-quenching (QQ) enzyme from *Chryseobacterium* sp. strain StRB126 that can effectively hydrolyze *N*-(3-oxododecanoyl) homoserine lactone (3OC12-HSL) and *N*-butanoyl-homoserine lactone (C4-HSL), the major signal molecules synthesized by *P. aeruginosa*. The exogenous addition of AidC to *P. aeruginosa* wild-type strain PAO1 cultures significantly reduced the total protease and elastase activities and the production of pyocyanin. In addition, the application of AidC resulted in thin and sparse biofilm formation. Later, we used a metagenomic-derived QQ enzyme, QQ-2, in combination with AidC to attenuate PAO1 virulence when the presence of a non-pseudomonad signal compound, autoinducer-2, aggravated it. These findings suggest that using a combined antimicrobial approach may lead to a more efficacious therapeutic intervention against *P. aeruginosa* PAO1 infection, as its behavior is modulated in the presence of intraspecies and interspecies signal compounds.

**One-Sentence Summary:**

In this work, the potential of dual enzymes was investigated to interfere with quorum sensing as a novel concept for reducing the virulence of *P. aeruginosa*, which is influenced by both intra species and interspecies communication.

## Graphical Abstract

**Graphical Abstract:**
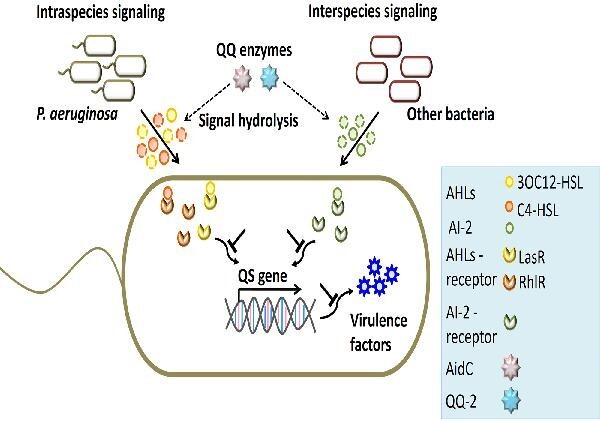
Dual enzyme-based quorum quenching strategy for reducing the virulence of *Pseudomonas aeruginosa*.

## Introduction

*Pseudomonas aeruginosa* is a common cause of nosocomial infections and a major pathogen in patients with cystic fibrosis and immunocompromised patients (Imperi et al., 2013; O'Loughlin et al., 2013; Taylor et al., 2022). As *P. aeruginosa* exhibits multidrug resistance to a broad spectrum of antibiotics, the strain is a high-priority target for the development of next-generation antibiotics that function through new mechanisms of action (Soukarieh et al., 2018; Taylor et al., 2022). *P. aeruginosa* pathogenicity is facilitated by several factors, including the ability to produce virulence factors such as exoenzymes, exotoxins, and pyocyanin, and the ability to form biofilms (Papaioannou et al., 2009; Pawar et al., 2015; Taylor et al., 2022). These characteristics are controlled by quorum sensing (QS), a cell density-reliant regulatory system dependent on the secretion of *N*-acylhomoserine lactones (AHLs) (Chadha et al., 2022; Flickinger et al., 2011; Taylor et al., 2022). Two AHL-mediated QS systems are found in *P. aeruginosa*, which consist of the Lux homologs LasRI and RhlRI. Two LuxI-type synthases, LasI and RhlI, produce 3OC12-HSL and C4-HSL, respectively (Boursier et al., 2020; Høyland-Kroghsbo et al., 2017; Soukarieh et al., 2018). These two signaling molecules are recognized by their cognate LuxR-type receptors, LasR and RhlR, and subsequently activate the expression of QS target genes (Boursier et al., 2020; Fong et al., 2018; O'Loughlin et al., 2013). In *P. aeruginosa*, swarming motility, biofilm maturation, and the expression of virulence factors such as exoproteases, hemolysins, exotoxin A, exoenzyme S, pyocyanin, cyanide, and the cytotoxic lectins PA-IL and PA-IIL are controlled by QS (Ahmed et al., 2021; Luo et al., 2017; Passador et al., 1993).

In addition to species-specific signals, such as AHL, researchers found that autoinducer-2 (AI-2) could affect the behaviors of *P. aeruginosa* (Duan et al., 2003; Li et al., 2022; Zhang et al., 2020). AI-2, which is a universal language in bacterial communication, can coordinate intraspecies and interspecies communication (Roy et al., 2013; Whiteley et al., 2017). It is encoded by the LuxS gene and can be produced by diverse genera (almost 50) and foster interspecies communication (Roy et al., 2013). While *P. aeruginosa* does not synthesize AI-2, it does sense AI-2 produced by the normal microflora of cystic fibrosis patients, leading to increased virulence factor expression and infection (Li et al., 2017; Zhang et al., 2020).

Since an active QS system is crucial for full pathogenicity, interfering with the QS systems of this pathogen may reduce virulence by preventing the release of harmful exoproducts and by inducing the development of abnormal biofilms that are more susceptible to conventional antibiotics. Thus, QS components are very attractive targets for the development of anti-infective therapy (Costerton et al., 1999; Whiteley et al., 2017). To date, inhibition of QS has been achieved by destabilizing the receptor protein for signal molecules (O'Loughlin et al., 2013; Storz et al., 2012; Thomann et al., 2016; Yang et al., 2012) and by degrading AHL signal compounds using acylase (Koch et al., 2014; Papaioannou et al., 2009; Park et al., 2005), lactonases (Chow et al., 2014; Ng et al., 2011; Pei & Lamas-Samanamud, 2014), and human paraoxonase PON (Aybey & Demirkan, 2016). PONs are originally described based on their ability to degrade organophosphates (OPs). Current studies have shown that PON enzymes also exhibit lactonase activities, are capable of hydrolyzing and inactivating AHLs, and are used for the disruption of biofilm formation in bacterial pathogens (Chow et al., 2014). In addition to PONs, the enzymes from the phosphotriesterase-like lactonase (PLL) family showed both paraoxonase activity toward OPs and lactonase activity on AHLs (Afriat et al., 2006; Hoque et al., 2017). The application of PLL enzymes, including GKL/GkaP from *Geobacillus kaustophilus*, SsoPox from *Sulfolobus solfataricus*, and *his-AhlA* from *Rhodococcus erythropolis*, showed promising results for attenuating the production of virulence factors as well as inhibiting the biofilm formation of the bacterial human pathogen (Chow et al., 2014; Marone et al., 2023; Ng et al., 2011). Recently, AHL-degrading enzymes have been found in other prokaryotes, including AidC, which is produced by *Chryseobacterium* sp. strain StRB126 (GenBank entry BAM28988, EC 3.1.1.81) (Wang et al., 2012). Analysis of protein sequence alignments revealed that AidC is homologous to other AHL lactonases found in the metallo-hydrolase/oxidoreductase superfamily, sharing a conserved dinuclear metal-binding motif (Mascarenhas et al., 2015). However, AidC has only ∼20 and ∼17% amino acid identity with AiiA (autoinducer inactivator A from *Bacillus* sp. 240B111) and AiiB (autoinducer inactivator B from *Agrobacterium tumefaciens* C58), respectively (Carlier et al., 2003; Dong et al., 2000; Mascarenhas et al., 2015). Steady-state kinetics revealed that AidC is the most efficient wild-type quorum-quenching (QQ) enzyme characterized to date (Mascarenhas et al., 2015; Wang et al., 2012). It shows high degradation activity against a broad set of AHLs, including 3OC12-HSL and C4-HSL, which indicates its potential for interfering with AHL-mediated communication in *P. aeruginosa*. To date, most QS inhibition strategies designed for *P. aeruginosa* have targeted species-specific signals, such as AHLs. Since this pathogen increases the expression of its virulence factor in response to AI-2 produced by other microflora (Duan et al., 2003; Li et al., 2022; Zhang et al., 2020), the methodologies to interrupt this cross-species signal may be essential for more effectively treating the infections caused by *P. aeruginosa*. Although an extensive study has been carried out for AHL-quenching compounds, only a very few AI-2 interfering mechanisms have been reported in detail thus far. These quenching mechanisms are mainly based on disrupting AI-2 synthesis and/or representing antagonistic small molecules (Roy et al., 2010b; Shen et al., 2006; Singh et al., 2006; Smith et al., 2009; Widmer et al., 2007). To date, a limited number of QQ enzymes that degrade or modify AI-2 have been reported (Roy et al., 2010a; Xavier et al., 2007). Recently, Schmitz's group identified a novel enzyme, QQ-2, and hypothesized that it reduces AI-2 to a QS-inactive AI-2 derivative (4-hydroxy-2,3-pentanedione-5-phosphate to 3,4,4-trihydroxy-2-pentanone-5-phosphate) (Weiland-Bräuer et al., 2016). In addition, the application of QQ-2 significantly inhibits AI-2-modulated biofilm formation of clinical *Klebsiella* isolates, which demonstrates its enormous potential for biotechnological application.

**Fig. 1.**
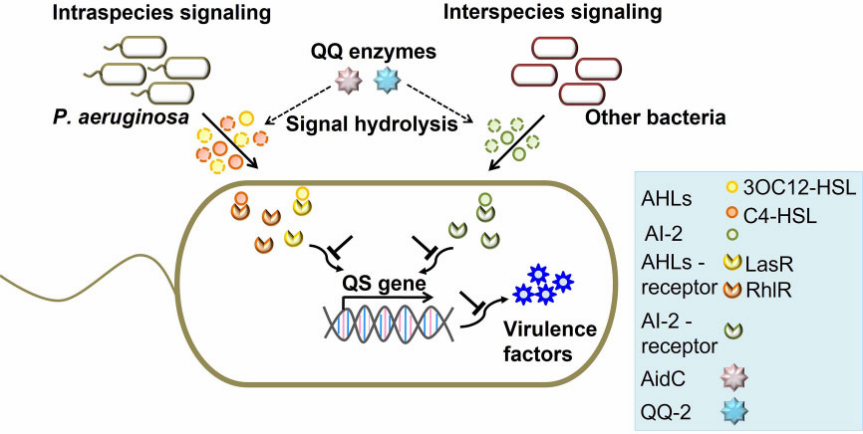
Schematic representation of *Pseudomonas aeruginosa* quorum sensing for virulence factor production at the intraspecies and interspecies levels. Simultaneous AidC and QQ-2 enzyme-based quorum quenching strategies.

In this study, we investigated the potential of the double enzyme to interfere with the *las-, rhl-*, and AI-2-mediated influence on QS-controlled virulence gene expression in *P. aeruginosa* (Fig. 1). With this aim, we used the highly active AHL lactonase AidC to degrade 3OC12-HSL and C4-HSL and the metagenome-derived enzyme QQ-2 to quench the AI-2 signaling molecule. The *in vitro* QQ activity of AidC and QQ-2 against *P. aeruginosa* was demonstrated by the decrease in protease, elastase, and pyocyanin production in the culture supernatant, which plays a critical role in host infection.

## Methods and Materials

### Chemicals

The substrates 3OC12-HSL and C4-HSL were purchased from Sigma-Aldrich Co. and Cayman Chemical Co., respectively. All other chemicals were analytical grade and were obtained from Sigma-Aldrich and Fisher Scientific.

### Bacterial Strains and Growth Conditions

Selected bacterial strains and plasmids used in this study are listed in Supplementary Table 1. *Pseudomonas aeruginosa* PAO1 (Huang et al., 2009) was routinely grown in Luria–Bertani broth (LB). *Escherichia coli* was grown in LB or 2YT medium (16 g/L tryptone, 1 g/L yeast extract, and 5 g/L sodium chloride). When needed, antibiotics were used at the following concentrations: ampicillin (100 µg/mL), kanamycin (50 µg/mL), and gentamicin (30 µg/mL).

The coding sequence for both AidC from *Chryseobacterium* sp. strain StRB126 and metagenome-derived QQ-2 (GenBank Accession No. JX870904) (Wang et al., 2012) were codon-optimized for expression in *E. coli*. A shuttle vector carrying the synthesized *aidC* gene and a protein expression vector, pMAL-C2X, were digested using the restriction enzymes BamHI and PstI. The resulting insert containing *aidC* was ligated into the expression vector using T_4_ DNA Ligase (New England Biolabs) to yield a protein expression plasmid encoding an N-terminal maltose-binding protein (MBP). Similarly, the EcoRI/HindIII-digested *qq-2* fragment was cloned into EcoRI/HindIII-digested pMAL-C2X to generate pMAL-QQ2.

### Expression and Purification of AidC and QQ-2 Proteins

To express AidC, the pMAL-AidC vector was used to transform *E. coli* BL21 (DE3) cells. The transformed cells were incubated at 37°C while being shaken in 2YT medium supplemented with 100 µg/mL ampicillin. When the culture OD_600_ value reached 0.6–0.8 absorbance units, expression of the MBP-AidC fusion proteins was induced by the addition of 0.3 mM isopropyl β-d-1-thiogalactopyranoside (IPTG). The 2YT medium was supplemented with 0.5 mM ZnSO_4_, and expression was continued for an additional 16–18 hr at 25°C after induction.

After cells were harvested by centrifugation at 12 400 *g*, pellets were resuspended in 1 × phosphate-buffered saline (PBS) (137 mM NaCl, 2.7 mM KCl, 10 mM Na_2_HPO_4_, and 1.8 mM KH_2_PO_4_) and lysed by using a French pressure cell with an internal cell pressure of 20 000 lb/in. The cell debris was removed by centrifugation, and the proteins were purified from the supernatant by affinity chromatography with amylose resin. The protein was eluted by 1 × PBS supplemented with 10 mM maltose. Protein samples were dialyzed in 1 × PBS at 4°C overnight to remove maltose prior to storage at  − 20°C. Protein purity and molecular weight were determined by SDS-PAGE (10%), followed by Coomassie blue staining. QQ-2 proteins were expressed and purified following a similar method as described for the AidC protein, except that ZnSO_4_ was added to the 2YT medium. Protein concentrations were determined with the Bradford assay kit (Bio-Rad, Hercules, CA, USA). The yields of total recombinant protein for AidC and QQ-2 were 18 mg/L and 7 mg/L, respectively.

### Enzymatic Assay

The kinetic measurements were monitored with a UV-2550 spectrophotometer (Shimadzu, Kyoto, Japan) at a constant temperature of 30°C. For stock solutions, the AHL substrate was dissolved in dimethyl sulfoxide (DMSO). The hydrolysis of 3OC12-HSL was monitored by using bicine buffer at PH 8.0 (2.5 mM bicine, 50 mM NaCl, 0.2 mM ZnCl2, and 0.5 mM m-cresol purple, 0.5% DMSO) over a concentration range of 0.2–2 mM. Hydrolysis was followed through the measurement of absorbance at 580 nm (ε = 2 923 M^−1^ cm^−1^) (Hiblot et al., 2013). The background hydrolysis with no enzyme was measured for substrate concentration and subtracted from the enzyme-catalyzed rates. The kinetic parameters were obtained by setting the acquired data to the Michaelis–Menten equation [V = S × E × *k_cat_*/(S + Km)] with GraphPad Prism software (Graphpad, San Diego, CA, USA), in which V is the initial velocity, E is the enzyme concentration, S is the substrate concentration, *k_cat_* is the turnover number, and Km is the Michaelis–Menten constant.

### *Chromobacterium violaceum* CV026-Based AHL Bioassay

Five microliters of an overnight LB culture of *C. violaceum* CV026 (Peng et al., 2018) were inoculated in 190 µL of fresh LB medium in a 96-well plate. To stimulate violacein synthesis, C4-HSL was added to each well at a final concentration of 10 µM. To evaluate whether AidC could modulate QS-dependent violacein production, AidC at a concentration of 100 µg/mL was added to each well, and the culture plates were then incubated in an orbital plate shaker at 220 rpm and 30°C. The assay in which 3OC12-HSL antagonized C4-HSL-mediated violacein synthesis in CV026 was performed as described previously (McClean et al., 1997). To carry out this, 5 mL of molten semisolid LB agar (0.3%, w/v) containing C4-HSL (final concentration, 10 µM) was seeded with 50 µL of an overnight LB culture of *C. violaceum* CV026 and poured immediately over the surface of prewarmed LB agar plates prepared in Petri dishes. When the overlaid agar solidified, wells were punched in the agar with a sterile cork borer. The wells were filled with either 3OC12-HSL at a concentration of 10 µM or 3OC12-HSL together with AidC. The Petri dishes were incubated in the upright position overnight at 30°C.

### Evaluating the Effect of AidC Protein on Growth of *P. Aeruginosa*

Overnight cultures of *P. aeruginosa* PAO1 inoculum were diluted in fresh LB broth to achieve a cell suspension OD_600__nm_ of 0.05 and aliquoted into a 96-well plate (Costar), with 200 µL in each well. AidC was added at different concentrations (100, 150, and 200 µg/mL) to the suspension and incubated at 30°C. At 6, 12, 18, and 24 hr, bacterial growth was monitored by measuring the optical density at 600 nm using a microplate reader (Spectra-Max M5).

### Detection of QS-Controlled Virulence Factors

#### Supernatant preparation

QS-controlled virulence factors present in the supernatants of *P. aeruginosa* cultures, such as LasA protease, LasB elastase, and pyocyanin, were detected. To do that, overnight cultures of *P. aeruginosa* PAO1 were adjusted to an OD_600__nm_ of 0.5 in LB medium and then incubated with appropriate concentrations (100–200 µg/mL) of AidC for 18 hr. After treatment, each group of culture samples was collected and centrifuged at 10 000 rpm for 15 min. The supernatants were filtered via passage through a syringe filter (0.22 µm, Millipore) and subsequently stored at –80°C or used immediately to detect the remaining virulence factors. To measure the amount of virulence factor production in the presence of AI-2, LB medium was supplemented with 20 nM AI-2. In the case of double enzyme treatment, 200 µg/mL and 30 µg/mL of AidC and QQ-2, respectively, were added to the AI-2-supplemented culture inoculum of *P. aeruginosa*.

#### Elastase assay

One milliliter of the elastase reaction mixture was composed of 10 mg of elastin-Congo red, 0.1 M Tris–HCl (pH 7.5), 1 mM CaCl_2_, and 100 µL of culture supernatant. The mixtures were incubated at 37°C for 2 hr with continuous rotation. After incubation, the samples were centrifuged at 12 000 rpm for 10 min to sediment the insoluble substrate. The absorbance of the supernatant was measured at 495 nm.

#### Protease assay

Azocasein degradation of culture supernatants was measured in reaction mixtures with a final volume of 1 mL composed of 0.33% (wt/vol) azocasein, 0.1 M Tris–HCl (pH 7.5), 1 mM CaCl_2_, and 100 µL of culture supernatant. The reaction mixture was incubated at 37°C for 30 min, and then the reaction was terminated by adding 500 µL of 10% (wt/vol) trichloroacetic acid. The absorbance of the supernatant was measured at 440 nm spectrophotometrically.

#### Pyocyanin assay

Pyocyanin was extracted from 1 mL of culture supernatant by chloroform extraction, followed by acidification. In brief, 1 mL of culture supernatant was added to 500 µL of chloroform and inverted 10 times to mix. The sample was centrifuged at 12 000 rpm for 5 min to separate the organic and aqueous phases. The aqueous phase was discarded, and 1 mL of 0.2 M HCl was added to acidify the pyocyanin within the organic phase. This sample was inverted 10 times to mix and then centrifuged at 12 000 rpm for 5 min before the absorbance was read at 520 nm.

### Scanning Electron Microscopy Analysis of *P. Aeruginosa* Biofilms

To perform structural analysis of the biofilm, an overnight culture of *P. aeruginosa* was diluted to an OD_600__nm_ of 0.05 and aliquoted into a 24-well plate containing 13-mm-diameter glass coverslips, with 1 mL in each well. Ten microliters of AidC (20 mg/mL) were added to each well, and the culture was grown for a total of 30 hr. After treatment, nonadherent cells from each sample were gently rinsed with sterile water for removal and subsequently fixed in 3% glutaraldehyde at 4°C overnight. The coverslips were rinsed 3 times with sterile water for 10 min each and then dehydrated in an ethanol gradient series (30%, 50%, 70%, 80%, and 90%) for 15 min per step. The samples were subsequently immersed in 100% ethanol (3 times for 10 min each) to prevent drying. Finally, all dehydrated samples were placed in a vacuum desiccator, coated with gold, and then observed using scanning electron microscopy (SEM).

### *Escherichia coli* Biofilm Formation Assay

The biofilm formation assay of *E. coli* was performed in a static environment in 96-well polystyrene microtiter plate. The 200 µL culture of *E. coli* cells at an optical density of 600 nm (OD_600_) of 0.05 was added to each well. The suspension was supplemented with different concentrations of QQ-2 (20 and 30 µg/mL) and incubated at 37°C without shaking. After 24 hr of cell growth, the biofilm formation was monitored and quantified using the crystal violet assay and measuring the absorbance at 590 nm as described previously (Li et al., 2017).

## Results and Discussion

### Expression and Purification of QQ Enzymes

To clone *aidC* and *qq-2* QQ genes, we used the pMAL-c2X vector to yield recombinant proteins N-terminally fused with MBP (Supplementary Fig. 1a and b). Heterologous expression of AidC and QQ-2 in DE3 cells using codon-optimized coding sequences led to good yields of soluble protein. The proteins were purified by maltose affinity chromatography, and the purity was analyzed by 10% SDS-PAGE. As expected, the results from the SDS-PAGE analysis revealed the expression of products that were approximately 78 and 70 kDa for fusion proteins AidC and QQ-2, respectively (Supplementary Fig. 1c and d).

### Determination of Kinetic Parameters of AidC for C4-HSL and 3-Oxo-C12-HSL

The previous characterization of AidC containing an N-terminal maltose-binding fusion protein showed high catalytic efficiency for a broader set of AHLs (Mascarenhas et al., 2015; Wang et al., 2012). However, the specificity constant of AidC for C4-HSL was reported in an earlier study (*k*_cat_/*K*_M_ 8.3 × 10^4^ M^−1^ s^−1^) and only the relative activity was assessed for 3OC12-HSL (Mascarenhas et al., 2015). In this study, the hydrolysis kinetics of AidC for 3OC12-HSL were determined by a plot of the substrate concentration and velocity. The *k*_cat_ and *K*_M_ values were calculated by fitting the data to the Michaelis‒Menten equation. We found that the enzyme exhibited high QQ lactonase activity with 3OC12-HSL, with a *k*_cat_/*K_M_*value of ∼ 4.4 × 10^5^ M^−1^ s^−1^ (Supplementary Table 2). Compared to other QQ enzymes, AidC is a much more efficient catalyst in terms of C4-HSL and 3OC12-HSL hydrolysis (Wang et al., 2012). Due to this property, this enzyme is more suitable for QQ application in *P. aeruginosa* than other homologs with higher catalytic efficiency for either C4-HSL or 3OC12-HSL.

### Effect of AidC on QS-Based Phenotype Modulation

To determine whether AidC can modulate the QS-based phenotype, we used the reporter strain *C. violaceum* (CV026) (Peng et al., 2018). Wild-type *C. violaceum* produces the characteristic purple pigment violacein, which is regulated by the *N*-hexanoyl-l-homoserine lactone (HHL)-dependent QS system (McClean et al., 1997). However, CV026 is a violacein-negative mutant in which pigment production can be restored by incubation with AHLs with acyl chain side chains from C_4_ to C_8_ in length (McClean et al., 1997). In this study, the incubation of CV026 in 96-well plates containing LB medium supplemented with C4-HSL induced violacein production (Supplementary Fig.2a-II). However, the addition of 100 µg/mL of AidC to LB medium completely inhibited C4-HSL-mediated violacein production in CV026 (Supplementary Fig. 2a-IV). This result suggested that AidC can effectively degrade C4-HSL and quench the C4-HSL-regulated QS phenomenon in a reporter strain.

In addition, it was previously reported that the presence of 3OC12-HSL in a culture medium containing CV026 and C4-HSL antagonized violacein production (McClean et al., 1997). To further evaluate whether AidC could alter this phenotype, we seeded C4-HSL and CV026 into the overlay agar and placed 3OC12-HSL in the wells. After overnight incubation, we observed colorless haloes surrounding the well on a purple background (Supplementary Fig. 2b). However, the well containing 3OC12-HSL and AidC did not cause a zone of antagonism with the purple background but rather led to a white zone of inhibition around the well (Supplementary Fig. 2c). This observation implied that AidC degraded both C4-HSL and 3OC12-HSL present in the agar plate. This is consistent with the substrate specificity of AidC, which shows strong catalytic efficiency for short- and long-chain AHLs (Wang et al., 2012).

### Effect of AidC on Virulence Factor Production in *P. Aeruginosa*

**Fig. 2.**
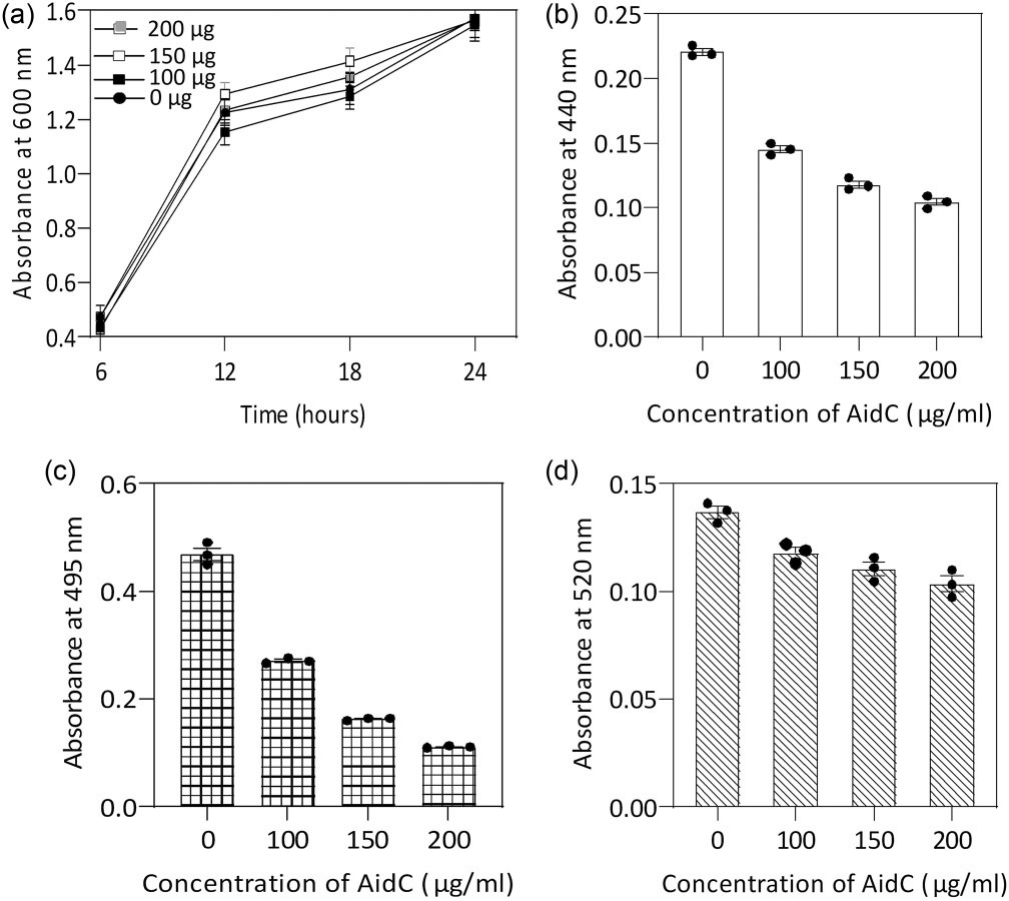
Effect of AidC on growth and quorum sensing in *Pseudomonas aeruginosa* PAO1. Cultures were incubated with 100, 150, or 200 µg/mL AidC. (a) AidC showed no effect on bacterial growth. (b) Culture supernatants were tested for protease activity, (c) elastase activity, (d), and pyocyanin levels.

As previously shown, AidC can degrade a broad range of AHLs and alter the QS phenomenon in reporter strains; thus, we hypothesized that it would be effective for disrupting the QS network in *P. aeruginosa*, which uses both C4-HSL and 3OC12-HSL. Exogenous pyocyanin production in *P. aeruginosa* is regulated by the C4-HSL-controlled QS system, whereas elastase and protease activities are regulated by the 3OC12-HSL-controlled QS system (Chow et al., 2014). To determine whether AidC can interfere with downstream signaling by *P. aeruginosa*, a culture of this bacterium was incubated with different concentrations of the enzyme (100, 150, and 200 µg/mL). As shown in Fig. 2a, the addition of the enzymes did not influence bacterial growth. However, the production of QS-relevant virulence factors was significantly reduced in a dose-dependent manner. When we monitored the proteolytic activity, we found that the addition of AidC to *P. aeruginosa* cultures significantly decreased the number of protease units detected in culture supernatants compared with the control culture (Fig. 2b). Additionally, at 18 hr, the level of elastase production was reduced by more than fourfold for the 200 µg enzyme treatment (Fig. 2c). These results indicated that the application of AidC can attenuate the production of *P. aeruginosa* PAO1 QS-associated virulence factors. However, at the same time points, the level of exogenous pyocyanin synthesis was slightly lower than the level for the control when 200 µg/mL of protein was supplied to the culture media (Fig. 2d). Besides AHL signal molecules, pseudomonas quinolone signal (PQS) plays a remarkable role in pyocyanin expression and regulation. In some cases, the degradation of the AHLs signal alone is not sufficient to completely diminish and block the QS activities, for example, AiiA lactonase can abolish and effectively quench the AHL signal molecules; however, it was found that it has a much lesser effect on the PQS system, which was also under *las* regulation (Fong et al., 2018).

### Effect of AidC on *P. Aeruginosa* Biofilm Architecture and Formation

**Fig. 3.**
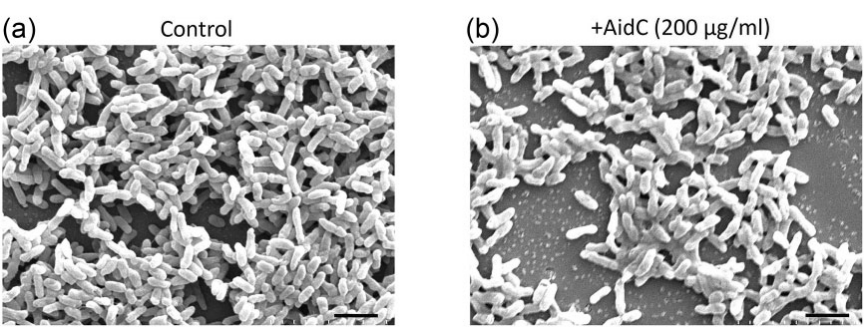
SEM images of *Pseudomonas aeruginosa* biofilms. (a) Biofilm is formed by *P. aeruginosa* without the addition of AHL lactonases. (b) *P. aeruginosa* biofilm in the presence of the AidC enzyme. Scale bar 5 µm. The biofilm structure analysis was analyzed by using MATLAB software.

To determine the effect of QQ lactonases on biofilm formation, the AidC enzyme was added to the log-phase culture of the wild-type *P. aeruginosa* PAO1 strain. We chose to use an AidC concentration of 200 µg/mL based on the observed maximum reduction in virulence factor production. After 30 hr of growth, SEM was used to assess the effect of lactonase treatment on the overall morphology and architecture of the *P. aeruginosa* biofilm. As shown in Fig. 3a and b, treatment with AidC caused a reduction in the biomass, thickness, and surface area of the biofilm. The quantitative analysis of SEM images revealed that the surface area for control and AidC treatment covered 78.14% and 59.02% of the overall image area, respectively (Supplementary Figs. 3 and 4). The biofilm structures for untreated *P. aeruginosa* cultures were significantly thicker and voluminous compared to cultures treated with AidC enzyme. Particularly, AidC-treated cells reduced biofilm volume by more than 16% compared to non-treated cells (Supplementary Figs. 4 and 5).

### Effect of QQ-2 on AI-2 Mediated Biofilm Formation

**Fig. 4.**
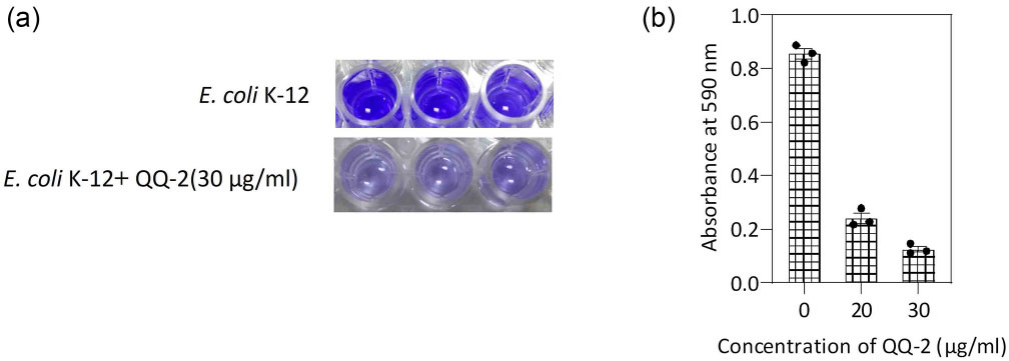
Inhibition of biofilm formation by MBP-QQ protein. (a) Images of the crystal violet assay for *Escherichia coli* K-12 cells grown with or without QQ-2 protein. (b) The absorbance at 590 nm was monitored in the suspension of *E. coli* K-12 cell culture in the presence or absence of QQ-2 protein.

AI-2 signaling plays an important role in *E. coli* biofilm formation (Weiland-Bräuer et al., 2016). To test the influence of the QQ-2 enzyme on AI-2-mediated biofilm formation, *E. coli* K-12 cells were cultured at 30°C for 24 hr in the absence or presence of MBP-QQ2 (20 and 30 µg/mL). As reported previously, biofilm formation was determined using crystal violet staining and measuring the absorbance at 590 nm (Li et al., 2017). As seen in Fig. 4a and b, AI-2-modulated biofilm formation of *E. coli* was efficiently inhibited even in the presence of a small amount of QQ-2 protein. These findings emphasize that the QQ-2 enzyme shows a strong inhibitory effect on AI-2-dependent biofilm formation.

### Effect of AI-2 and Double Enzyme Treatment on Virulence Factor Production

**Fig. 5.**
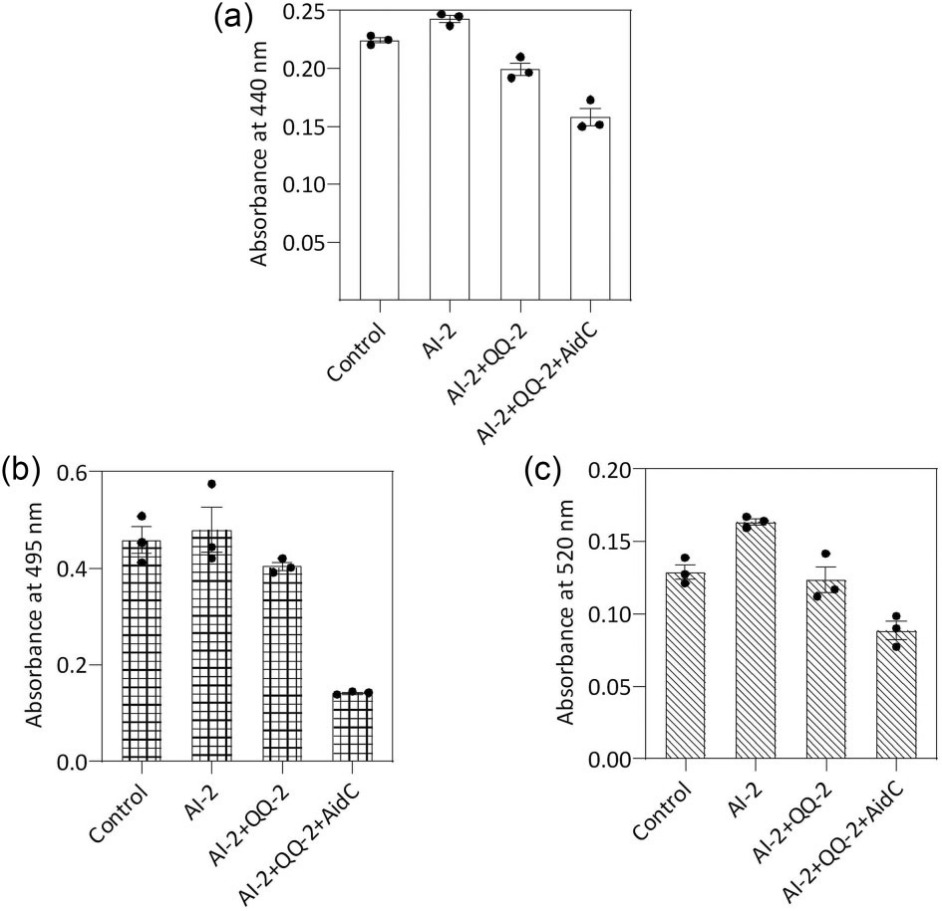
The effect of autoinducer-2 (AI-2) and double enzyme treatment on quorum sensing (QS)-modulated virulence factor production in *Pseudomonas aeruginosa* PAO1. Cultures were incubated with 20 nm AI-2 and 200 µg/mL and 30 µg/mL AidC and QQ-2 proteins, respectively. (a) Culture supernatants were tested for protease activity, (b) elastase activity, and (c) pyocyanin levels.

The signaling molecule of the LuxS-dependent quorum-sensing system is termed AI-2. AI-2 is considered an interspecies signaling molecule (Roy et al., 2013). However, *P. aeruginosa* PAO1 cannot produce AI-2 because a *luxS* gene homolog has not been identified in its genome (Duan et al., 2003). Nevertheless, recent studies have revealed that AI-2 promotes pathogenesis mediated by *P. aeruginosa* (Li et al., 2017). In this study, when we investigated the effect of AI-2 in terms of protease, elastase, and pyocyanin, it was observed that adding 20 nM to the culture medium could positively influence virulence factor production. The AI-2-supplemented culture shows higher protease activity and pyocyanin production compared with cells grown without AI-2 (Fig. 5a and c), which is congruent with the findings of previous studies. On the other hand, a slight effect of AI-2 was observed for elastase activity in *P. aeruginosa* cultures (Fig. 5c). Since interspecies signaling molecules, such as AI-2, modulate QS-controlled virulence factor production, QS interference-based combined therapeutic approaches that can block inter- and intraspecies signaling would be effective for preventing this pathogen. To this end, we added AidC along with the QQ-2 enzyme at concentrations of 200 µg/mL and 30 µg/mL in the log-phase culture of *P. aeruginosa*. Although the addition of QQ-2 protein exerted a slight impact on the inhibition of virulence factor production, it eventually reduced the yield of protease, elastase, and pyocyanin when used in combination with AidC enzyme (Fig. 5a–c).

### Effect of AI-2 *P. Aeruginosa* Biofilm Architecture and Formation

**Fig. 6.**
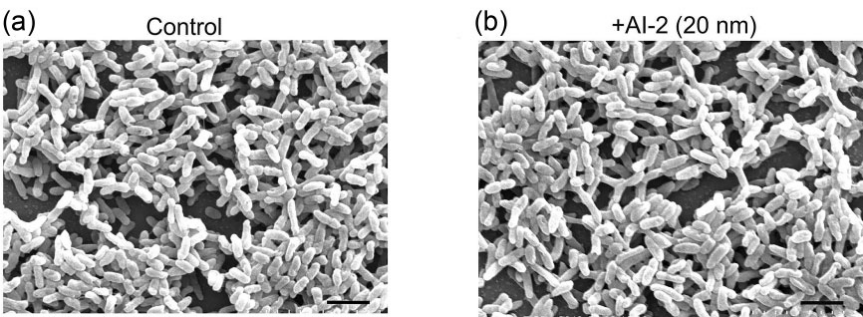
SEM images of *Pseudomonas aeruginosa* biofilms. (a) Biofilm formed by *P. aeruginosa* without the addition of autoinducer-2 (AI-2). (b) *P. aeruginosa* biofilm in the presence of 20 nM AI-2. Scale bar 5 µm.

As exogenous AI-2 acts as a *P. aeruginosa* virulence factor production promoter, we subsequently investigated its impact on biofilm formation. However, we observed no significant change in biofilm structure between the cells growing with or without AI-2. Highly ordered and stable biofilm architecture, including microchanneling necessary to maintain the flow of nutrients, was observed for AI-2-treated and untreated cultures of *P. aeruginosa* (Fig. 6a and b).

## Conclusion

In this study, we demonstrated the potential of dual enzymes to interfere with QS as a novel concept for the prevention of *P. aeruginosa* pathogenesis, which is influenced by intraspecies and interspecies communication. We chose the highly active QQ enzymes AidC and QQ-2 to impede the *las, rhl*, and AI-2-mediated influence on QS-controlled virulence gene expression in this pathogen. The application of two enzymes can significantly attenuate the virulence of *P. aeruginosa*, which is aggravated by the addition of the AI-2 precursor. Presumably, this beneficial effect is due to lower signal molecule levels, which inhibit bacterial communication and restrict the expression of genes related to virulence production. In addition, AidC enzyme treatment results in thinner biofilms with altered architecture. Compared to untreated biofilms, biofilms treated with lactonase are less ordered and have an apparent lack of cohesive biofilm structure. This type of alteration in biofilm could increase the susceptibility of bacteria toward antibiotic treatments, which further suggests the potentiation of traditional antibiotics. In the translation of this work to clinical application, the potential of the developed system can be investigated in the intricate cellular system, where parameters obtained from the *in vitro* study would be valuable to predict the *in vivo* system.

## Supplementary Material

kuad028_Supplemental_File

## References

[bib1] Afriat L. , Roodveldt C., Manco G., Tawfik D. S. (2006). The latent promiscuity of newly identified microbial lactonases is linked to a recently diverged phosphotriesterase. Biochemistry, 45(46), 13677–13686. 10.1021/bi061268r 17105187

[bib2] Ahmed S. A. K. S. , Rudden M., Elias S. M., Smyth T. J., Marchant R., Banat I. M., Dooley J. S. G. (2021). *Pseudomonas aeruginosa* PA80 is a cystic fibrosis isolate deficient in RhlRI quorum sensing. Scientific Reports, 11(1), 5729. 10.1038/s41598-021-85100-0 33707533 PMC7970962

[bib3] Aybey A. , Demirkan E. (2016). Inhibition of *Pseudomonas aeruginosa* biofilm formation and motilities by human serum paraoxonase (Hpon1). AIMS Microbiology, 2(4), 388–401. 10.3934/MICROBIOL.2016.4.388 26654051

[bib4] Boursier M. E. , Combs J. B., Blackwell H. E. (2020). Correction to “*N*-acyl l-homocysteine thiolactones are potent and stable synthetic modulators of the RhlR quorum sensing receptor in *Pseudomonas aeruginosa*”. ACS Chemical Biology, 15(6), 1718–1718. 10.1021/acschembio.0c00331 32395993 PMC10804912

[bib5] Carlier A. , Uroz S., Smadja B., Fray R., Latour X., Dessaux Y., Faure D. (2003). The Ti plasmid of *Agrobactetium tumefaciens* harbors an attM-paralogous gene, aiiB, also encoding *N*-acyl homoserine lactonase activity. Applied and Environmental Microbiology, 69(8), 4989–4993. 10.1128/AEM.69.8.4989-4993.2003 12902298 PMC169067

[bib6] Chadha J. , Harjai K., Chhibber S. (2022). Revisiting the virulence hallmarks of *Pseudomonas aeruginosa*: A chronicle through the perspective of quorum sensing. Environmental Microbiology, 24(6), 2630–2656. 10.1111/1462-2920.15784 34559444

[bib7] Chow J. Y. , Yang Y., Tay S. B., Chua K. L., Yew W. S. (2014). Disruption of biofilm formation by the human pathogen *Acinetobacter baumannii* using engineered quorum-quenching lactonases. Antimicrobial Agents and Chemotherapy, 58(3), 1802–1805. 10.1128/AAC.02410-13 24379199 PMC3957888

[bib8] Costerton J. W. , Stewart P. S., Greenberg E. P. (1999). Bacterial biofilms: A common cause of persistent infections. Science, 284(5418), 1318–1322. 10.1126/science.284.5418.1318 10334980

[bib9] Dong Y. H. , Xu J. L., Li X. Z., Zhang L. H. (2000). AiiA, an enzyme that inactivates the acylhomoserine lactone quorum-sensing signal and attenuates the virulence of *Erwinia carotovora*. Proceedings of the National Academy of Sciences of the USA, 97(7), 3526–3531. 10.1073/pnas.97.7.3526 10716724 PMC16273

[bib10] Duan K. , Dammel C., Stein J., Rabin H., Surette M. G. (2003). Modulation of *Pseudomonas aeruginosa* gene expression by host microflora through interspecies communication. Molecular Microbiology, 50(5), 1477–1491. 10.1046/j.1365-2958.2003.03803.x 14651632

[bib11] Flickinger S. T. , Copeland M. F., Downes E. M., Braasch A. T., Tuson H. H., Eun Y. J., Weibel D. B. (2011). Quorum sensing between *Pseudomonas aeruginosa* biofilms accelerates cell growth. Journal of the American Chemical Society, 133(15), 5966–5975. 10.1021/ja111131f 21434644 PMC3076519

[bib12] Fong J. , Zhang C., Yang R., Boo Z. Z., Tan S. K., Nielsen T. E., Givskov M., Liu X. W., Bin W., Su H., Yang L. (2018). Combination therapy strategy of quorum quenching enzyme and quorum sensing inhibitor in suppressing multiple quorum sensing pathways of *P. aeruginosa*. Scientific Reports, 8(1), 1155. 10.1038/s41598-018-19504-w 29348452 PMC5773576

[bib13] Hiblot J. , Gotthard G., Elias M., Chabriere E. (2013). Differential active site loop conformations mediate promiscuous activities in the lactonase SsoPox. PLoS ONE, 8(9), e75272. 10.1371/journal.pone.0075272 24086491 PMC3781021

[bib14] Hoque M. A. , Zhang Y., Chen L., Yang G., Khatun M. A., Chen H., Hao L., Feng Y. (2017). Stepwise loop insertion strategy for active site remodeling to generate novel enzyme functions. ACS Chemical Biology, 12(5), 1188–1193. 10.1021/acschembio.7b00018 28323400

[bib15] Høyland-Kroghsbo N. M. , Paczkowski J., Mukherjee S., Broniewski J., Westra E., Bondy-Denomy J., Bassler B. L. (2017). Quorum sensing controls the *Pseudomonas aeruginosa* CRISPR-Cas adaptive immune system. Proceedings of the National Academy of Sciences of the USA, 114(1), 131–135. 10.1073/pnas.1617415113 27849583 PMC5224376

[bib16] Huang J. , Xu Y., Zhang H., Li Y., Huang X., Ren B., Zhang X. (2009). Temperature-dependent expression of *phzM* and its regulatory genes *lasI* and *ptsP* in rhizosphere isolate *Pseudomonas* sp. strain M18. Applied and Environmental Microbiology, 75(20), 6568–6580. 10.1128/AEM.01148-09 19717631 PMC2765144

[bib17] Imperi F. , Massai F., Facchini M., Frangipani E., Visaggio D., Leoni L., Bragonzi A., Visca P. (2013). Repurposing the antimycotic drug flucytosine for suppression of *Pseudomonas aeruginosa* pathogenicity. Proceedings of the National Academy of Sciences of the USA, 110(18), 7458–7463. 10.1073/pnas.1222706110 23569238 PMC3645532

[bib18] Koch G. , Nadal-Jimenez P., Reis C. R., Muntendam R., Bokhove M., Melillo E., Dijkstra B. W., Cool R. H., Quax W. J. (2014). Reducing virulence of the human pathogen *Burkholderia* by altering the substrate specificity of the quorum-quenching acylase PvdQ. Proceedings of the National Academy of Sciences of the USA, 111(4), 1568–1573. 10.1073/pnas.1311263111 24474783 PMC3910591

[bib19] Li H. , Li X., Ai Q., Tan L. (2022). Autoinducer-2 promotes *Pseudomonas aeruginosa* PAO1 acute lung infection via the IL-17A pathway. Frontiers in Microbiology, 13. 10.3389/fmicb.2022.948646 PMC9404534 36033859

[bib20] Li H. , Li X., Song C., Zhang Y., Wang Z., Liu Z., Wei H., Yu J. (2017). Autoinducer-2 facilitates *Pseudomonas aeruginosa* PAO1 pathogenicity *in vitro* and *in vivo*. Frontiers in Microbiology, 8, 1944. 10.3389/fmicb.2017.01944 29089927 PMC5651085

[bib21] Luo J. , Dong B., Wang K., Cai S., Liu T., Cheng X., Lei D., Chen Y., Li Y., Kong J., Chen Y. (2017). Baicalin inhibits biofilm formation, attenuates the quorum sensing-controlled virulence and enhances *Pseudomonas aeruginosa* clearance in a mouse peritoneal implant infection model. PLoS ONE, 12(4), e0176883. 10.1371/journal.pone.0176883 28453568 PMC5409170

[bib22] Marone M. , Porzio E., Lampitella E. A., Manco G. (2023). A mesophilic phosphotriesterase-like lactonase shows high stability and proficiency as quorum quenching enzyme. Chemico-Biological Interactions, 383, 110657. 10.1016/j.cbi.2023.110657 37573927

[bib23] Mascarenhas R. , Thomas P. W., Wu C. X., Nocek B. P., Hoang Q. Q., Liu D., Fast W. (2015). Structural and biochemical characterization of AidC, a quorum-quenching lactonase with atypical selectivity. Biochemistry, 54(28), 4342–4353. 10.1021/acs.biochem.5b00499 26115006 PMC4681436

[bib24] McClean K. H. , Winson M. K., Fish L., Taylor A., Chhabra S. R., Camara M., Daykin M., Lamb J. H., Swift S., Bycroft B. W., Stewart G. S. A. B., Williams P. (1997). Quorum sensing and *Chromobacterium violaceum*: Exploitation of violacein production and inhibition for the detection of *N*-acylhomoserine lactones. Microbiology, 143(12), 3703–3711. 10.1099/00221287-143-12-3703 9421896

[bib25] Ng F. S. W. , Wright D. M., Seah S. Y. K. (2011). Characterization of a phosphotriesterase-like lactonase from *Sulfolobus solfataricus* and its immobilization for disruption of quorum sensing. Applied and Environmental Microbiology, 77(4), 1181–1186. 10.1128/AEM.01642-10 21183649 PMC3067241

[bib26] O'Loughlin C. T. , Miller L. C., Siryaporn A., Drescher K., Semmelhack M. F., Bassler B. L. (2013). A quorum-sensing inhibitor blocks *Pseudomonas aeruginosa* virulence and biofilm formation. Proceedings of the National Academy of Sciences of the USA, 110(44), 17981–17986. 10.1073/pnas.1316981110 24143808 PMC3816427

[bib27] Papaioannou E. , Wahjudi M., Nadal-Jimenez P., Koch G., Setroikromo R., Quax W. J. (2009). Quorum-quenching acylase reduces the virulence of *Pseudomonas aeruginosa* in a *Caenorhabditis elegans* infection model. Antimicrobial Agents and Chemotherapy, 53(11), 4891–4897. 10.1128/AAC.00380-09 19721066 PMC2772301

[bib28] Park S. Y. , Kang H. O., Jang H. S., Lee J. K., Koo B. T., Yum D. Y. (2005). Identification of extracellular *N*-acylhomoserine lactone acylase from a *Streptomyces* sp. and its application to quorum quenching. Applied and Environmental Microbiology, 71(5), 2632–2641. 10.1128/AEM.71.5.2632-2641.2005 15870355 PMC1087586

[bib29] Passador L. , Cook J. M., Gambello M. J., Rust L., Iglewski B. H. (1993). Expression of *Pseudomonas aeruginosa* virulence genes requires cell-to-cell communication. Science, 260(5111), 1127–1130. 10.1126/science.8493556 8493556

[bib30] Pawar V. , Komor U., Kasnitz N., Bielecki P., Pils M. C., Gocht B., Moter A., Rohde M., Weiss S., Häussler S. (2015). *In vivo* efficacy of antimicrobials against biofilm-producing *Pseudomonas aeruginosa*. Antimicrobial Agents and Chemotherapy, 59(8), 4974–4981. 10.1128/AAC.00194-15 26055372 PMC4505208

[bib31] Pei R. , Lamas-Samanamud G. R. (2014). Inhibition of biofilm formation by T7 bacteriophages producing quorum-quenching enzymes. Applied and Environmental Microbiology, 80(17), 5340–5348. 10.1128/AEM.01434-14 24951790 PMC4136088

[bib32] Peng H. , Ouyang Y., Bilal M., Wang W., Hu H., Zhang X. (2018). Identification, synthesis and regulatory function of the *N*-acylated homoserine lactone signals produced by *Pseudomonas chlororaphis* HT66. Microbial Cell Factories, 17(1), 9. 10.1186/s12934-017-0854-y 29357848 PMC5776774

[bib33] Roy V. , Fernandes R., Tsao C. Y., Bentley W. E. (2010a). Cross species quorum quenching using a native AI-2 processing enzyme. ACS Chemical Biology, 5(2), 223–232. 10.1021/cb9002738 20025244

[bib34] Roy V. , Meyer M. T., Smith J. A. I., Gamby S., Sintim H. O., Ghodssi R., Bentley W. E. (2013). AI-2 analogs and antibiotics: A synergistic approach to reduce bacterial biofilms. Applied Microbiology and Biotechnology, 97(6), 2627–2638. 10.1007/s00253-012-4404-6 23053069

[bib35] Roy V. , Smith J. A. I., Wang J., Stewart J. E., Bentley W. E., Sintim H. O. (2010b). Synthetic analogs tailor native AI-2 signaling across bacterial species. Journal of the American Chemical Society, 132(32), 11141–11150. 10.1021/ja102587w 20698680

[bib36] Shen G. , Rajan R., Zhu J., Bell C. E., Pei D. (2006). Design and synthesis of substrate and intermediate analogue inhibitors of *S*-ribosylhomocysteinase. Journal of Medicinal Chemistry, 49(10), 3003–3011. 10.1021/jm060047g 16686542

[bib37] Singh V. , Shi W., Almo S. C., Evans G. B., Furneaux R. H., Tyler P. C., Painter G. F., Lenz D. H., Mee S., Zheng R., Schramm V. L. (2006). Structure and inhibition of a quorum sensing target from *Streptococcus pneumoniae*. Biochemistry, 45(43), 12929–12941. 10.1021/bi061184i 17059210 PMC2517848

[bib38] Smith J. A. I. , Wang J., Nguyen-Mau S. M., Lee V., Sintim H. O. (2009). Biological screening of a diverse set of AI-2 analogues in *Vibrio harveyi* suggests that receptors which are involved in synergistic agonism of AI-2 and analogues are promiscuous. Chemical Communications, 45(45), 7033. 10.1039/b909666c 19904385

[bib39] Soukarieh F. , Williams P., Stocks M. J., Cámara M. (2018). *Pseudomonas aeruginosa* quorum Sensing systems as drug discovery targets: Current position and future perspectives. Journal of Medicinal Chemistry 61(23), 10385–10402. 10.1021/acs.jmedchem.8b00540 29999316

[bib40] Storz M. P. , Maurer C. K., Zimmer C., Wagner N., Brengel C., De Jong J. C., Lucas S., Müsken M., Häussler S., Steinbach A., Hartmann R. W. (2012). Validation of PqsD as an anti-biofilm target in *Pseudomonas aeruginosa* by development of small-molecule inhibitors. Journal of the American Chemical Society, 134(39), 16143–16146. 10.1021/ja3072397 22992202

[bib41] Taylor I. R. , Jeffrey P. D., Moustafa D. A., Goldberg J. B., Bassler B. L. (2022). The PqsE active site as a target for small molecule antimicrobial agents against *Pseudomonas aeruginosa*. Biochemistry, 61(17), 1894–1903. 10.1021/acs.biochem.2c00334 35985643 PMC9454246

[bib42] Thomann A. , De Mello Martins A. G. G., Brengel C., Empting M., Hartmann R. W. (2016). Application of dual inhibition concept within looped autoregulatory systems toward antivirulence agents against *Pseudomonas aeruginosa* infections. ACS Chemical Biology, 11(5), 1279–1286. 10.1021/acschembio.6b00117 26882081

[bib43] Wang W. Z. , Morohoshi T., Someya N., Ikeda T. (2012). Aidc, a novel *N*-acylhomoserine lactonase from the potato root-associated cytophaga-flavobacteria-bacteroides (CFB) group bacterium *Chryseobacterium* sp. strain StRB126. Applied and Environmental Microbiology, 78(22), 7985–7992. 10.1128/AEM.02188-12 22941089 PMC3485932

[bib44] Weiland-Bräuer N. , Kisch M. J., Pinnow N., Liese A., Schmitz R. A. (2016). Highly effective inhibition of biofilm formation by the first metagenome-derived AI-2 quenching enzyme. Frontiers in Microbiology, 7, 7985. 10.3389/fmicb.2016.01098 PMC4942472 27468282

[bib45] Whiteley M. , Diggle S. P., Greenberg E. P. (2017). Progress in and promise of bacterial quorum sensing research. Nature, 551(7680), 313–320. 10.1038/nature24624 29144467 PMC5870893

[bib46] Widmer K. W. , Soni K. A., Hume M. E., Beier R. C., Jesudhasan P., Pillai S. D. (2007). Identification of poultry meat-derived fatty acids functioning as quorum sensing signal inhibitors to autoinducer-2 (AI-2). Journal of Food Science, 72(9), M363–M368. 10.1111/j.1750-3841.2007.00527.x 18034729

[bib47] Xavier K. B. , Miller S. T., Lu W., Kim J. H., Rabinowitz J., Pelczer I., Semmelhack M. F., Bassler B. L. (2007). Phosphorylation and processing of the quorum-sensing molecule autoinducer-2 in enteric bacteria. ACS Chemical Biology, 2(2), 128–136. 10.1021/cb600444h 17274596

[bib48] Yang Y. X. , Xu Z. H., Zhang Y. Q., Tian J., Weng L. X., Wang L. H. (2012). A new quorum-sensing inhibitor attenuates virulence and decreases antibiotic resistance in *Pseudomonas aeruginosa*. Journal of Microbiology, 50(6), 987–993. 10.1007/s12275-012-2149-7 23274985

[bib49] Zhang L. , Li S., Liu X., Wang Z., Jiang M., Wang R., Xie L., Liu Q., Xie X., Shang D., Li M., Wei Z., Wang Y., Fan C., Luo Z. Q., Shen X. (2020). Sensing of autoinducer-2 by functionally distinct receptors in prokaryotes. Nature Communications, 11(1), 5371. 10.1038/s41467-020-19243-5 PMC7584622 33097715

