# Supplementary material for: Reducing the virulence of Pseudomonas aeruginosa by using multiple quorum-quenching enzymes
Source: J Ind Microbiol Biotechnol. 2023 Sep 20;50(1):kuad028. doi: 10.1093/jimb/kuad028 (PMC10536470; doi:10.1093/jimb/kuad028)
Supplement: kuad028_Supplemental_File [file kuad028_supplemental_file.docx]

Supporting information for

**Reducing the virulence of *Pseudomonas aeruginosa* by using multiple quorum quenching enzymes**

Mst Afroza Khatun^1^, Md Anarul Hoque ^2^, Mattheos Koffas^2^, Yan Feng^1^,*

^1^State Key Laboratory of Microbial Metabolism, School of Life Sciences and Biotechnology, Shanghai Jiao Tong University, Shanghai 200240, China. ^2^Department of Chemical and Biochemical Engineering, Center for Biotechnology and Interdisciplinary Studies, Rensselaer Polytechnic Institute, Troy, New York 12180, USA.

* Corresponding author, Email: yfeng2009@sjtu.edu

**Table S1.** Bacterial strain and plasmid were used in this study.

| **Strains/Plasmids** | **Description** | | **Source or reference** |
| --- | --- | --- | --- |
| **Strains** | |  |  |
| *E. coli* | |  |  |
| DH5α | | F^-^ φ80*lacZ*ΔM15 Δ(lacZYA-argF) U169 recA1 endA1 hsdR17 (r_k_^-^ m_k_^+^) phoA supE44 λ^-^ thi-1 gyrA96 relA1 | Invitrogen^b^ |
| BL21(DE3) | | recA1 endA1 gyrA96 thi-1 hsdR17 (r_k_^-^ m_k_^+^ ) supE44 relA1 lac (F′ proAB lacI^q^ZΔM15 Tn10 [Tet ^r^ ]) | Stratagene |
| K12 MG1655 | |  | ATTC |
| *P. aeruginosa* PAO1 | | Wild type | (Huang et al., 2009) |
| *C. violaceum* CV026 | |  | (Peng et al., 2018) |
| **Plasmids** | |  |  |
| pMAL-c2X | | Cloning vector to make MBP fusions; Amp^r^ | NEB |
| pMAL-QQ2 | | containing *qq-2* gene | this study |
| pMAL-AidC | | containing *aidC* gene from StRB126 | this study |


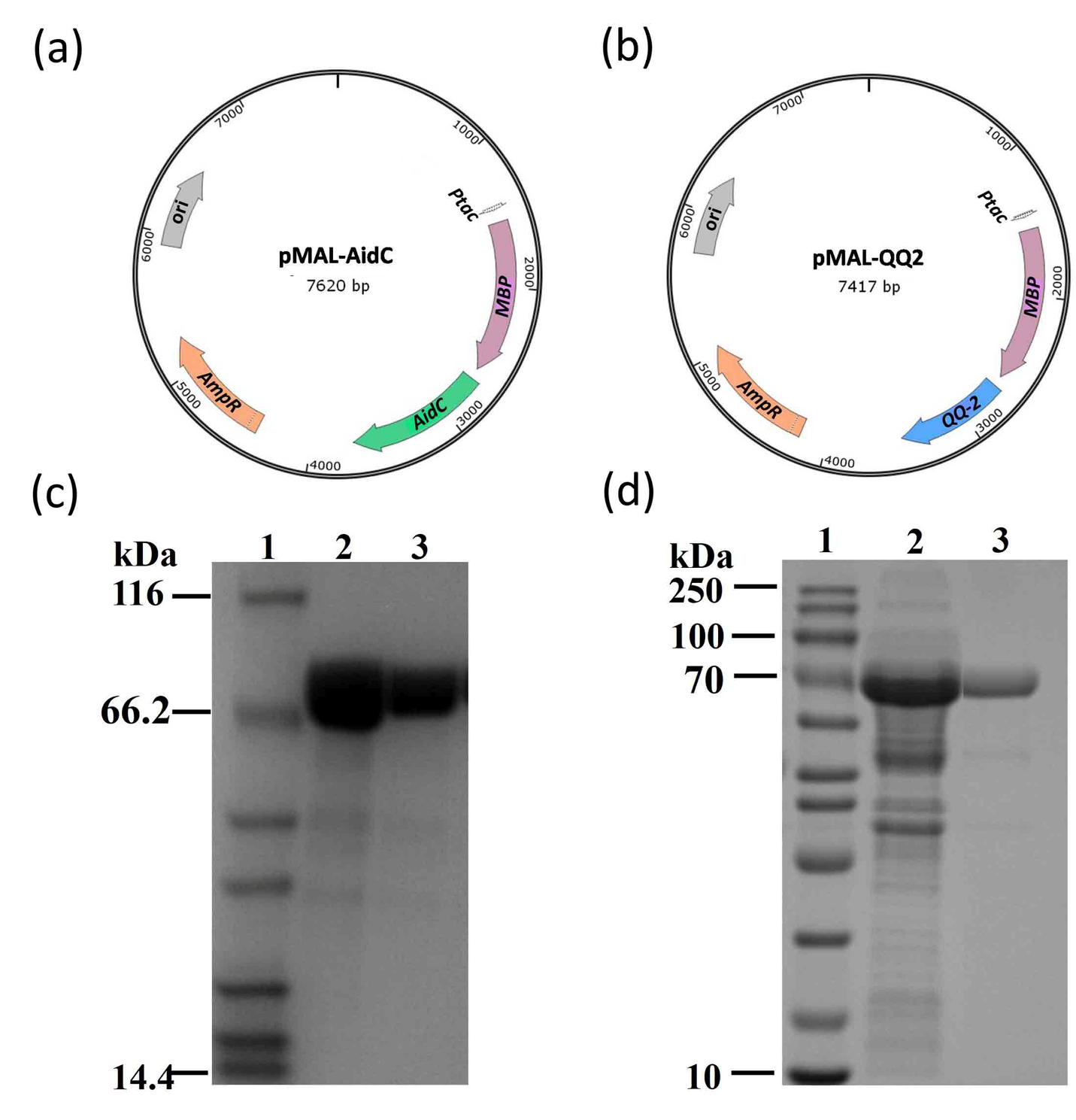


**Fig. S1.** Cloning and purification of AidC and QQ-2 proteins. (a) plasmid map of pMAL-AidC. (b) plasmid map of pMAL-QQ2. (c) SDS-PAGE of purified AidC protein (lane 2, 15µg of AidC and lane 3, 8µg of AidC protein. (d) SDS-PAGE of purified QQ-2 protein, lane 1 cell lysate; 2 purified protein.

**Table S2.** Steady-state kinetic parameters of AidC for C4-HSL and 3OC12-HSL


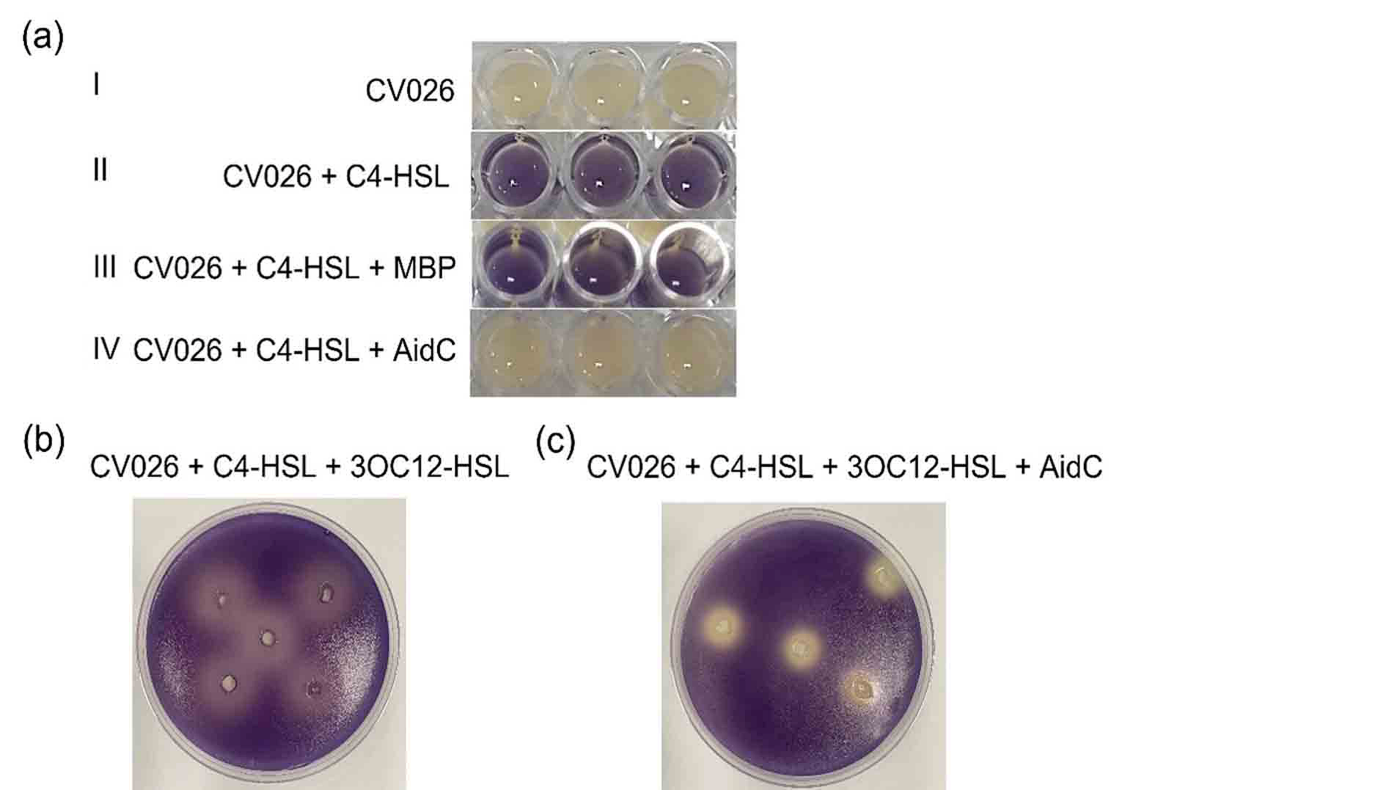


| **Substrate** | **K_M_ (mM)** | ***k*_cat_ (s^-1^)** | ***k*_cat/_K_M_ (M^-1^ s^-1^)** |
| --- | --- | --- | --- |
| C4-HSL  3OC12-HSL | 0.47±0.06  0.56 ±0.01 | 39±2  250±18 | 8.3× 10^4^(Mascarenhas et al.,2015)    4.4 × 10^5^ (This work) |

**Fig. S2.** *Chromobacterium violaceum* CV026-based AHL bioassay. CV026 is a violacein-negative mutant (a-I). For the induction of violacein production C4-HSL was added to each well at a final concentration of 10 µM (a-II). The effect of 50µg of MBP in the LB medium containing CV026+C4-HSL (a-III). The addition of 100µg of AidC in the LB medium inhibited the C4-HSL-mediated violacein production in CV026 (a-IV). 3-oxo-C_12_-HSL based antagonism was observed for C4-HSL mediated violacein synthesis in CV026. Well cut into the LB soft agar plate containing C4-HSL and CV026 and placed either 3OC_12-_HSL only (b) or both 3-oxo-C_12-_HSL and AidC to examine their quorum-quenching effects (c).


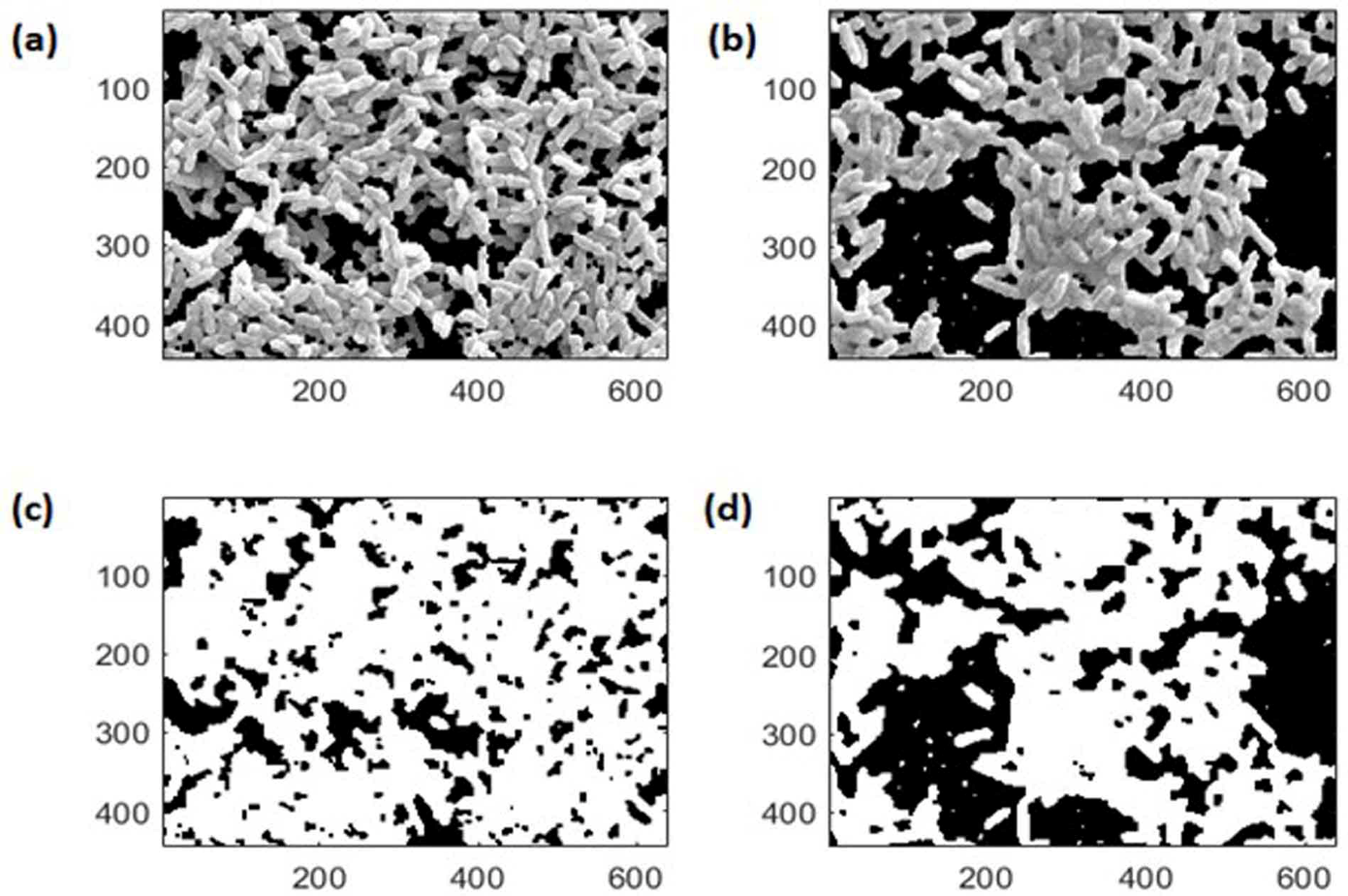


**Fig. S3.** Control (a) and AidC treated cells (b) scans after background noise removal. (c) and (d) can be represented as the binary masks that indicate the surface area covered, which are 78.14% and 59.02% of the overall image area, respectively. Hence, the surface area has been reduced at least by 20%. To effectively analyze the pixel distribution, we performed background removal using the Segment Anything model by Facebook, available in Huggingface as an API (<https://huggingface.co/spaces/angelasnpang/segment-anything-ui>). After background removal, from Fig. S3, we can see that the histograms obtain Gaussian-like shapes over the range of 50-240.


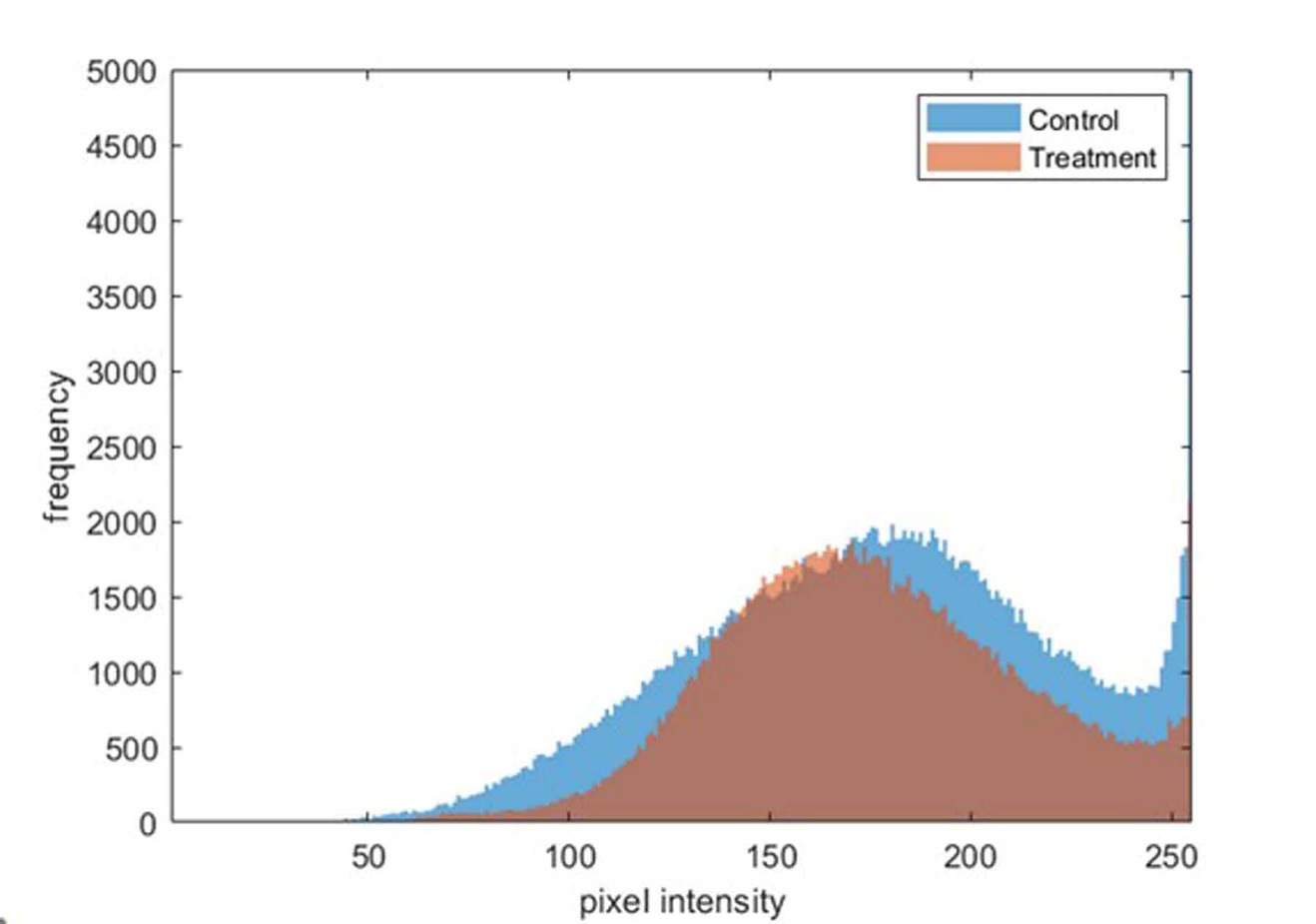


**Fig. S4.** Histogram analysis of control (a) and AidC treated culture treatment with their respective pixel intensity. It can be seen that the pixel intensity distributions have Gaussian-like shapes for both control and AidC-treated cells.

Here, to get an estimation of the thickness, we model the two histograms using split normal distributions. Split normal distributions are normal distributions except the standard deviations are different for the two sides. Then, we could consider the effective thickness to be the sum of the two standard deviations. The equation for the split normal curve would be:


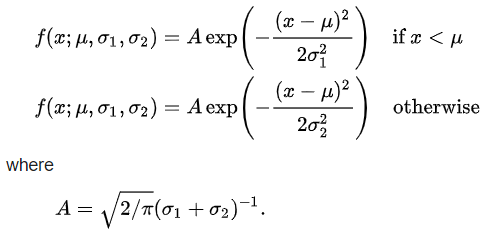


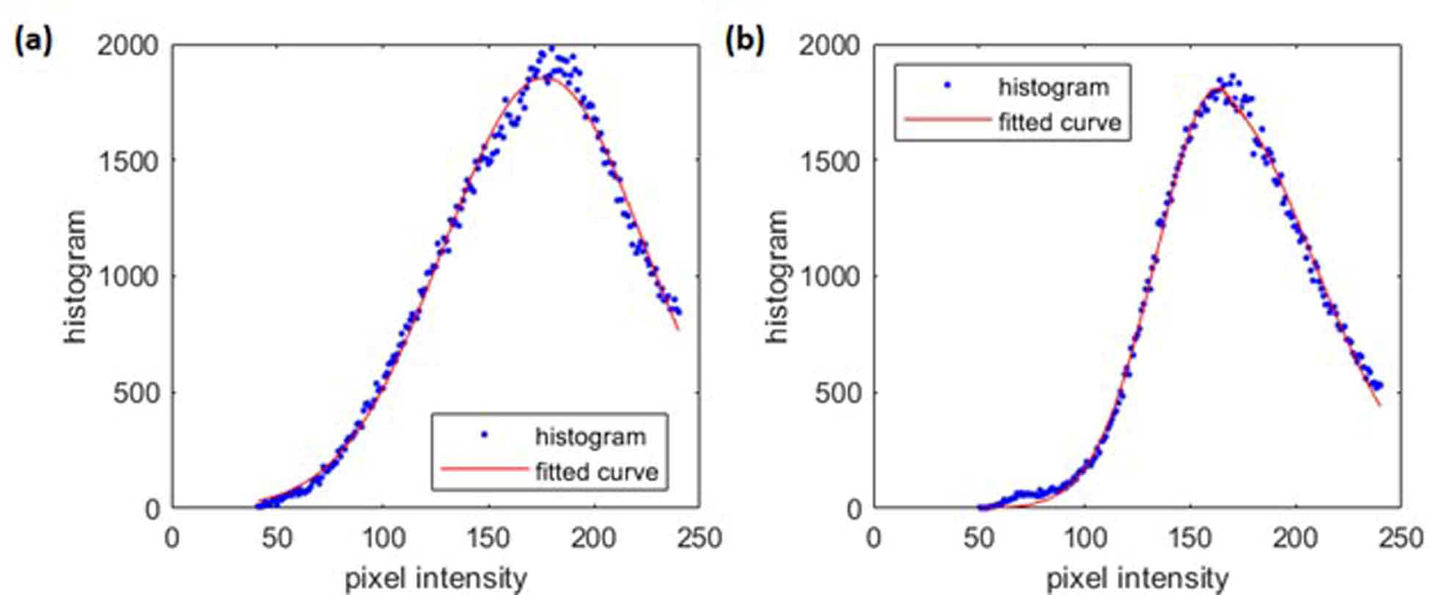


**Fig. S5.** Fitted curves for (a) control and (b) treatment histograms.

After fitting the curves, we found for control, the coefficients are (with a 95% confidence interval),

A = 1855 (1841, 1870)

μ = 176.4 (175.9, 176.9)

σ_1_ = 67.55 (66.8, 68.31)

σ_2_ = 67.53 (66.76, 68.27)

Similarly, for treatment,

A = 1810 (1794, 1826)

μ = 163.5 (162.6, 164.3)

σ_1_ = 41.43 (40.57, 42.29)

σ_2_ = 67.46 (64.76, 70.17)

Hence, for control, the total standard distribution is σ_C_ = 135.08 and for treatment, it is σ_T_ = 108.89. If we consider the total thickness of the biofilm to have the pixel intensity range [0-255], then the effective thickness would be 52.97% and 42.70%, respectively. This indicates that the biofilm has experienced a minimum thickness reduction of 10%. If we consider the thickness of the control biofilm to be 100%, then the treatment thickness should be at 80.61%. Undoubtedly, the original thickness is much less than that.

If we consider the volume to be the multiplication of surface area and thickness, then for control the volume would be 78.14% x 52.97% = 41.39%, whereas for treatment it would be 59.02% x 42.70% = 25.20%.
